# CELSR1 Promotes Neuroprotection in Cerebral Ischemic Injury Mainly through the Wnt/PKC Signaling Pathway

**DOI:** 10.3390/ijms21041267

**Published:** 2020-02-13

**Authors:** Li-Hong Wang, Geng-Lin Zhang, Xing-Yu Liu, Ai Peng, Hai-Yuan Ren, Shu-Hong Huang, Ting Liu, Xiao-Jing Wang

**Affiliations:** 1 Department of Cell Biology, School of Basic Medical Sciences, Shandong University, Jinan 250012, Shandong, China; wanglihong1202@163.com (L.-H.W.); 201714954@mail.sdu.edu.cn (X.-Y.L.); pengai1007@163.com (A.P.); ren15993562826@163.com (H.-Y.R.); liuting@sdu.edu.cn (T.L.); 2 Key Laboratory for Biotech-Drugs Ministry of Health and Key Laboratory for Rare & Uncommon Diseases of Shandong Province, Shandong Medicinal Biotechnology Center, Shandong First Medical University & Shandong Academy of Medical Sciences, Jinan 250062, Shandong, China; zgelin@163.com; 3 Institute of Basic Medicine, Shandong First Medical University & Shandong Academy of Medical Sciences, Jinan 250062, Shandong, China; shuhonghuang@sdu.edu.cn; 4 Advanced Medical Research Institute, Shandong University, Jinan 250012, Shandong, China

**Keywords:** CELSR1, neurogenesis, angiogenesis, stroke, Wnt/PKC pathway

## Abstract

Cadherin epidermal growth factor (EGF) laminin G (LAG) seven-pass G-type receptor 1 (CELSR1) is a member of a special subgroup of adhesion G protein-coupled receptors. Although *Celsr1* has been reported to be a sensitive gene for stroke, the effect of CELSR1 in ischemic stroke is still not known. Here, we investigated the effect of CELSR1 on neuroprotection, neurogenesis and angiogenesis in middle cerebral artery occlusion (MCAO) rats. The mRNA expression of *Celsr1* was upregulated in the subventricular zone (SVZ), hippocampus and ischemic penumbra after cerebral ischemic injury. Knocking down the expression of *Celsr1* in the SVZ with a lentivirus significantly reduced the proliferation of neuroblasts, the number of CD31-positive cells, motor function and rat survival and increased cell apoptosis and the infarct volume in MCAO rats. In addition, the expression of p-PKC in the SVZ and peri-infarct tissue was downregulated after ischemia/ reperfusion. Meanwhile, in the dentate gyrus of the hippocampus, knocking down the expression of *Celsr1* significantly reduced the proliferation of neuroblasts; however, it had no influence on motor function, cell apoptosis or angiogenesis. These data indicate that CELSR1 has a neuroprotective effect on cerebral ischemia injury by reducing cell apoptosis in the peri-infarct cerebral cortex and promoting neurogenesis and angiogenesis, mainly through the Wnt/PKC pathway.

## 1. Introduction

Stroke is the second-most fatal disease worldwide [1], and approximately 87% of stroke cases are ischemic stroke triggered by blood flow blockage within major cerebral arteries. Currently, the only FDA-approved treatment for acute ischemic stroke is intravenous recombinant tissue plasminogen activator (tPA) [2], but the majority of patients cannot benefit from this agent due to its narrow treatment time window and association with hemorrhagic complications [3,4]. Therefore, it is necessary to find other more effective therapies for cerebral ischemia.

Cerebral ischemic can induce spontaneous neurological repair processes, including neurogenesis and angiogenesis [5,6]. Adult neurogenesis occurs mainly in the subventricular zone (SVZ) of the lateral ventricles and the subgranular zone (SGZ) in the hippocampal dentate gyrus (DG) [7,8]. In the middle cerebral artery occlusion (MCAO) model, neural stem cells (NSCs) in the SVZ can generate many neuroblasts and migrate to the ischemic penumbra, improving neurological functional recovery [9]. Cerebral ischemia injury can stimulate the expression of endogenous vascular-related factors, thereby promoting the rapid proliferation of vascular endothelial cells, and migrate to the damaged areas to form new blood vessels, providing nutrients and oxygen for the neurons in the ischemic penumbra [5,10].

CELSR1 is an adhesion G protein-coupled receptor [11,12,13,14]. *Celsr1* was identified as a susceptibility gene for ischemic stroke in Japanese individuals by a genome-wide association study [15,16]. Recent evidence has suggested that CELSR1 regulates the direction of dendrite initiation sites [17,18]. In vitro, CELSR1 is a positive regulator of endothelial cell migration and angiogenesis [19]. In addition, CELSR1 is also a key component of the noncanonical Wnt/planar cell polarity (PCP) pathway, and it is involved with Fzd3, Fzd6, Dvl1, Dvl2 and Vangl2 in the Wnt/PCP pathway [20,21]. In this decade, more studies have indicated that the noncanonical Wnt/PCP pathway also regulates endothelial cell proliferation and angiogenesis [22,23,24]. Thus far, the role of CELSR1 in cerebral ischemia is still unclear. To address these questions, we administered lentiviral microinjections to MCAO rats to knock down the expression of Celsr1 to assess the role of CELSR1 in neuroprotection, neurogenesis and angiogenesis in cerebral ischemia in an MCAO model.

## 2. Results

### 2.1. The Expression Level of Celsr1 Increased Significantly in the Ischemic SVZ and DG

**Figure 1:**
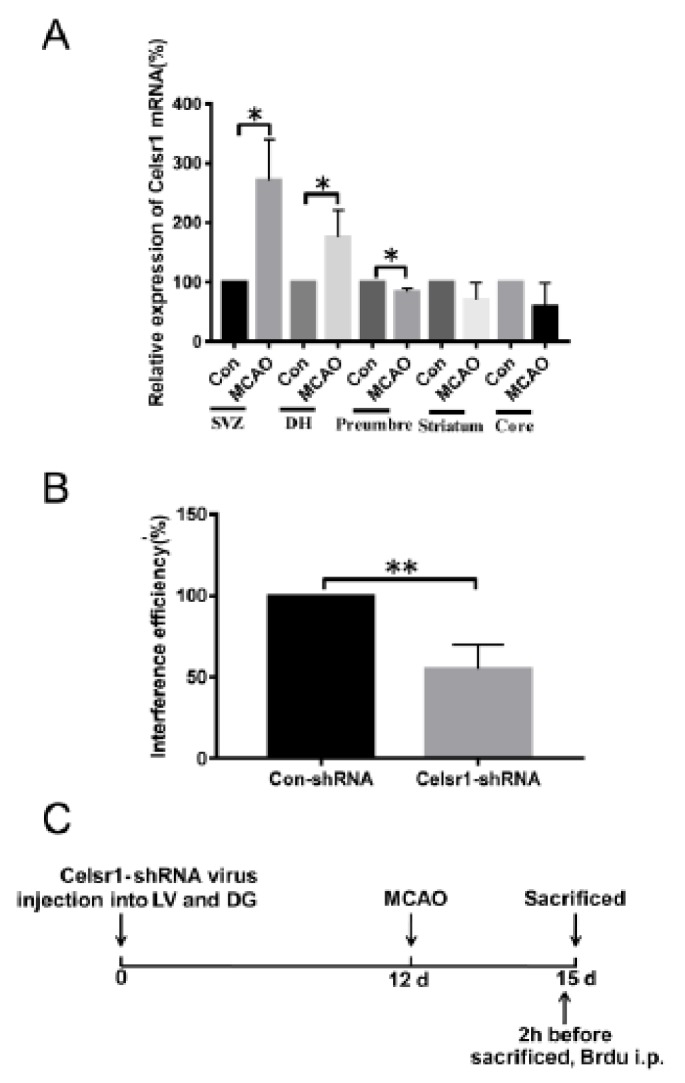
The expression of *Celsr1* in the different brain areas of middle cerebral artery occlusion (MCAO) rats. (**A**) After 2 h of ischemia/24 h of reperfusion, q-PCR was conducted in the subventricular zone (SVZ), dentate gyrus (DG), ischemic penumbra, ischemic core and ischemic cortex (*n* = 3 in each group). * *p* < 0.05. (**B**) The interference efficiency of the *Celsr1*-shRNA lentivirus in 293 cells was tested by q-PCR (*n* = 3, the experiment was repeated 3 times). ** *p* < 0.01. (**C**) Experimental scheme of pretreatment with the *Celsr1*-shRNA lentivirus.

To test whether CELSR1 participates in the process of cerebral ischemia, we investigated the mRNA expression of *Celsr1* by quantitative RT-PCR after 2 h of ischemia/22 h of reperfusion. Compared to that in the sham group (100%), the mRNA expression of *Celsr1* in the MCAO group was significantly increased in the SVZ and DG (SVZ: 271.4% ± 48.69%, *p* = 0.0180; DG: 175.9% ± 26.26%, *p* = 0.0446, Figure 1A), decreased in the ischemic penumbra (83.74% ± 3.635%, *p* = 0.0110, Figure 1A), and showed no obvious change in the ischemic core (58.81% ± 28.14%, *p* = 0.2809, Figure 1A) and striatum (71.53% ± 15.89%, *p* = 0.1477, Figure 1A). These results suggest that CELSR1 may play a role in cerebral ischemic injury.

### 2.2. A Celsr1-shRNA Lentivirus Was Constructed and Microinjected into the Brain

To identify the roles of CELSR1 in cerebral ischemic injury, a *Celsr1*-shRNA lentivirus with green fluorescent protein (GFP) was constructed. To test the efficiency of *Celsr1* knockdown, HEK293T cells were transfected with the *Celsr1*-shRNA lentivirus and a control lentivirus. According to the results of quantitative RT-PCR, the interference efficiency of the *Celsr1*-shRNA lentivirus reached approximately 50% compared to that of the control lentivirus (54.88% ± 8.69% vs. 100%, *p* = 0.0016, Figure 1B). The *Celsr1*-shRNA lentivirus and control lentivirus were microinjected into the lateral ventricle and DG of rats. After 12 days, rats underwent MCAO. The rats were sacrificed three days after MCAO (Figure 1C).

### 2.3. Knockdown of Celsr1 in the SVZ Accelerated Brain Injury Induced by Ischemia/Reperfusion 

**Figure 2:**
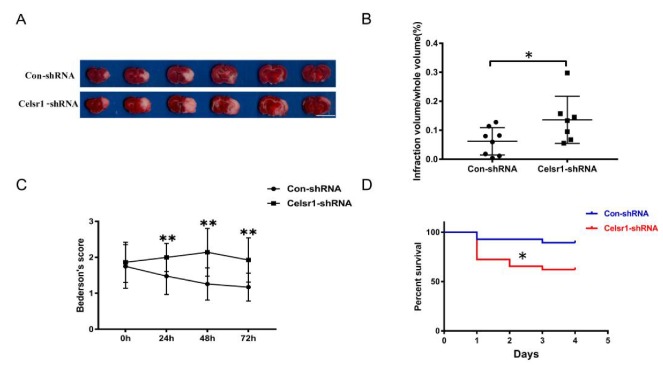
*Celsr1* knockdown in the SVZ accelerated brain injury. (**A**) Triphenyltetrazolium chloride (TTC) staining was used to show the infarct volume in the brain sections. The representative images were placed in order from the anterior to the posterior portion of the brain, from left to right. Scale bar = 10 mm. (**B**) The infarct volume was quantified by TTC staining. *Celsr1* knockdown significantly increased the infarct volume in the MCAO rats (Con-shRNA group, *n* = 8; *Celsr1*-shRNA group, *n* = 7). * *p* < 0.05. (**C**) *Celsr1* knockdown significantly increased the Bederson score (*n* = 5 per group, ** *p* < 0.01). (**D**) The mortality rate was significantly increased in the *Celsr1*-shRNA group. * *p* < 0.05.

The *Celsr1*-shRNA lentivirus and control lentivirus were microinjected into the SVZ of rats twelve days before MCAO. After 2 h of ischemia/70 h of reperfusion, the rats were sacrificed. Then, the cerebral infarct volume was assessed. The group treated with *Celsr1*-shRNA lentivirus had a significantly larger infarct volume than the control group (0.06% ± 0.02% vs. 0.14% ± 0.03%, *p* = 0.047 Figure 2A,B). Neurological deficits were assessed after 22 h, 46 h and of reperfusion following 2 h of ischemia. The *Celsr1*-shRNA lentivirus group showed a significantly higher Bederson score than that of the control group at different time points, peaking after 2 h of ischemia/46 h of reperfusion (1.26 ± 0.09 vs. 2.2 ± 0.17, *p* < 0.001, Figure 2C). Interestingly, we found that most of the rats that died after MCAO were in the *Celsr1*-shRNA group. Therefore, we calculated the mortality of rats in the control group and *Celsr1*-shRNA group after MCAO. Compared to that of the control group, the mortality rate caused by ischemia/reperfusion injury in the *Celsr1*-shRNA lentivirus group increased three-fold (Figure 2D). This shows that *Celsr1* knockdown increased the brain infarct volume and neurological deficit score and then led to animal death. This suggests that CELSR1 has a protective effect on cerebral ischemic injury.

### 2.4. Celsr1 Knockdown in the SVZ Increased the Percentage of Apoptotic Cells in the Peri-infarct Cerebral Cortex

**Figure 3:**
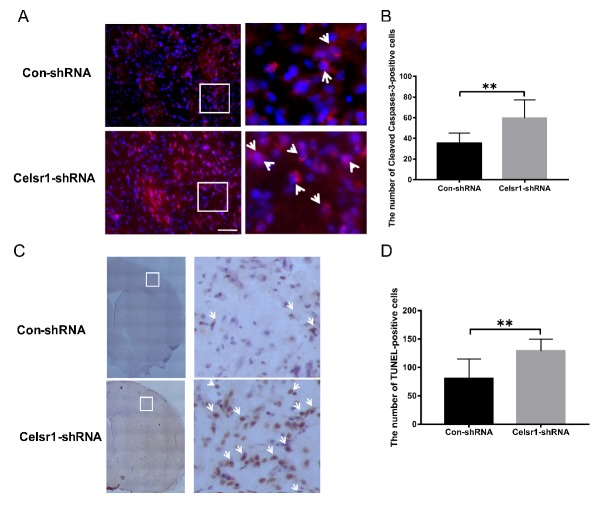
*Celsr1* knockdown in the SVZ increased the number of apoptotic cells in the ischemic penumbra. (**A**) Cleaved caspase-3-positive cells (red) were identified by immunohistochemical staining, and 4,6-diamidino-2-phenylindole (DAPI) (blue) was used to label the nuclei. The insets white box to show photomicrographs of the cells at higher magnification. Arrows point to caspase-3-positive cells. Scale bar = 45 μm. (**B**) Quantitative analysis of the number of caspase-3-positive cells in the ischemic penumbra (Con-shRNA group, *n* = 5; *Celsr1*-shRNA group, *n* = 4). (**C**) TdT-mediated dUTP-biotin nick end labeling (TUNEL) staining of apoptotic cells in the ischemic penumbra. The insets white box to show photomicrographs of the ischemic penumbra at higher magnification. Arrows point to TUNEL-positive cells. Scale bar = 45 μm. (**D**) Quantitative analysis of TUNEL-positive cells in the ischemic penumbra (Con-shRNA group, *n* = 5; *Celsr1*-shRNA group, *n* = 4). ** *p* < 0.01.

The mechanism of the neuroprotective effect of CELSR1 was explored. Can *Celsr1* knockdown promote cell apoptosis in the peri-infarct cerebral cortex to increase brain injury? Cleaved caspase-3 and TdT-mediated dUTP-biotin nick end labeling (TUNEL) staining were applied to brain sections. Compared with that in the control group, the number of caspase-3-positive cells in the peri-infarct cerebral cortex was increased significantly in the SVZ of the *Celsr1*-shRNA lentivirus group (59.72 ± 4.14 vs. 35.5 ± 2.40, *p* = 0.0001, Figure 3A,B). Both the cleaved caspase-3-positive cells and TUNEL-positive cells were apoptotic cells. There were more TUNEL-positive cells in the *Celsr1*-shRNA lentivirus group than in the control group (129.10 ± 5.34 vs. 80.58 ± 9.90, *p* = 0.0001, Figure 3C,D). These results indicate that *Celsr1* knockdown in the SVZ can increase the number of apoptotic cells in the peri-infarct cerebral cortex.

### 2.5. Celsr1 Knockdown in the SVZ Inhibited Neurogenesis and Angiogenesis after Cerebral Ischemia

**Figure 4:**
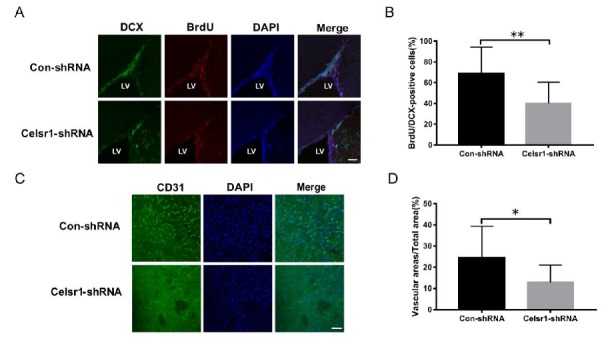
*Celsr1* knockdown in the SVZ reduced neurogenesis and angiogenesis. (**A**) *Celsr1* knockdown in the SVZ significantly reduced the number of BrdU/DCX-positive cells. Scale bar = 45 μm. (**B**) Quantitative analysis of BrdU/DCX-positive cells in the SVZ (Con-shRNA group, *n* = 5; *Celsr1*-shRNA group, *n* = 4). ** *p* < 0.01. (**C**) CD31 staining (green) in the ischemic penumbra. DAPI (blue) was used to label the nuclei. Scale bar = 45 μm. (**D**) Quantitative analysis of CD31-positive cells upon *Celsr1* knockdown in the SVZ (Con-shRNA group, *n* = 5; *Celsr1*-shRNA group, *n* = 5). * *p* < 0.05.

Another mechanism by which CELSR1 exerts neuroprotection was explored by investigating neurogenesis and angiogenesis in the SVZ. BrdU (a marker that labels newborn cells) and DCX (an immature neuronal marker) were used to identify neurogenesis in the SVZ. There were fewer BrdU/nestin-positive cells in the SVZ of the *Celsr1*-shRNA lentivirus group than in the SVZ of the control group (68.91% ± 5.41% vs. 40.00% ± 5.29%, *p* = 0.0008, Figure 4A,B). CD31 (a marker of vascular endothelial cells) was used to evaluate angiogenesis around the SVZ. The *Celsr1*-shRNA lentivirus group had a lower CD31-positive vascular area ratio around the SVZ (12.88% ± 2.11%) than the control group (24.61% ± 3.68%, *p* = 0.0109, Figure 4C,D). These results indicate that *Celsr1* knockdown inhibits neurogenesis and angiogenesis in the SVZ.

### 2.6. Celsr1 Knockdown in the SVZ Suppressed the Wnt/PKC Signaling Pathway after Cerebral Ischemia

**Figure 5:**
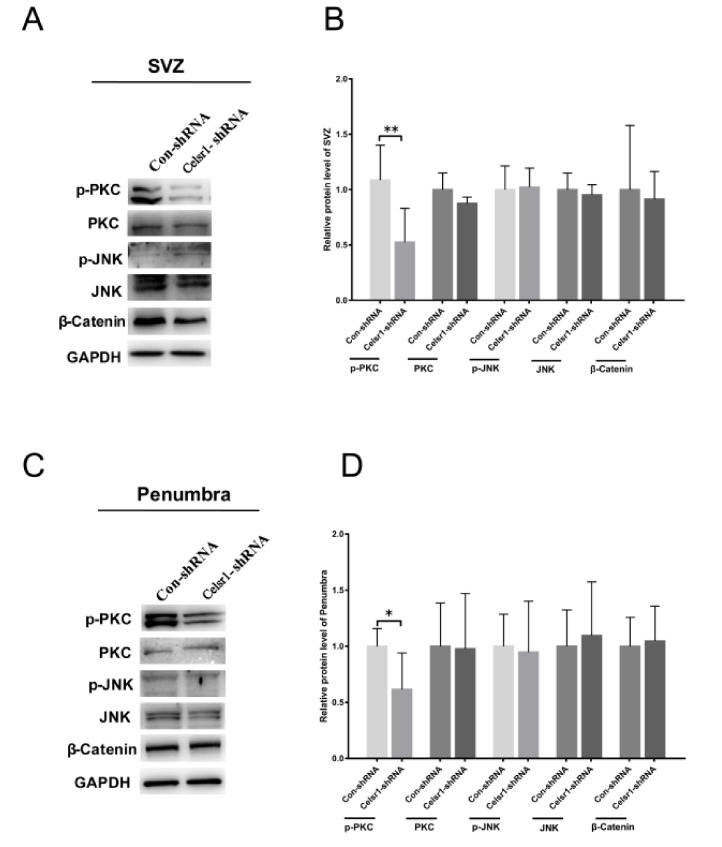
CELSR1 regulates neurogenesis after cerebral ischemia through the Wnt/PKC signaling pathway. (**A**) *Celsr1* knockdown in the SVZ significantly reduced the level of p-PKC, but the levels of p-JNK and β-catenin did not change. (**B**) The protein levels in the SVZ were quantified relative to the level of GAPDH. (**C**) The level of p-PKC was significantly reduced in the ischemic penumbra. (**D**) The protein levels in the penumbra were relative to the level of GAPDH, and three independent experiments were performed. The reported data represent the mean of the three experiments, Student’s *t*-test, * *p* < 0.05, ** *p* < 0.01 (Con-shRNA group, *n* = 6; *Celsr1*-shRNA group, *n* = 6).

To deeply clarify the neuroprotective mechanism of CELSR1 in cerebral ischemic injury, tissues of the SVZ and ischemic penumbra from the control group and *Celsr1*-shRNA group were harvested. The results showed that the expression level of p-PKC was significantly reduced in two brain areas in the *Celsr1*-shRNA group compared to the control group (SVZ: 0.52 ± 0.12 vs. 1.09 ± 0.11, *p* = 0.004, Figure 5A,B; ischemic penumbra: 0.62 ± 0.13 vs. 1.00 ± 0.07, *p* = 0.0396, Figure 5C,D), but the expression levels of PKC, p-JNK, JNK and β-catenin were not obviously different in the *Celsr1*-shRNA group compared to the control group. These results indicate that the neuroprotection exerted by CELSR1 in cerebral ischemic injury may occur through the Wnt/PKC signaling pathway.

### 2.7. Celsr1 Knockdown in the DG Had no Influence on Cell Apoptosis in the Peri-infarct Cerebral Cortex or Neurological Deficit Scores 

**Figure 6:**
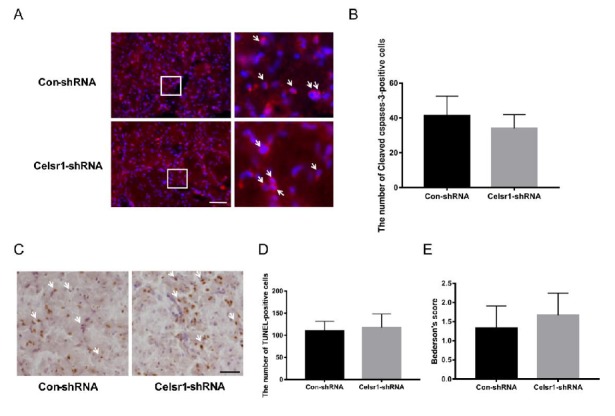
*Celsr1* knockdown in the DG had no effect on the number of apoptotic cells in the ischemic penumbra. (**A**) Cleaved caspase-3 staining (red) in the ischemic penumbra, DAPI (blue) was used to label the nuclei. The insets white box to show photomicrographs of the cells at higher magnification. Arrows point to caspase-3-positive cells. Scale bar = 45 μm. (**B**) The number of cleaved caspase-3-positive cells was not different, as determined by quantitative analysis after *Celsr1* knockdown in the DG. (**C**) TUNEL staining was used to show apoptotic cells. Arrows point to TUNEL-positive cells. Scale bar = 45 μm. (**D**) No difference was found in the number of TUNEL-positive cells by quantitative analysis (*n* = 3 per group). (**E**) The Bederson score showed no change after *Celsr1* knockdown in the DG (*n* = 3 per group).

To deeply test the neuroprotection exerted by CELSR1 in cerebral ischemic injury, the *Celsr1*-shRNA lentivirus and control lentivirus were microinjected into the DG of the hippocampus twelve days before MCAO. Immunostaining of cleaved caspase-3 and TUNEL staining were used to identify cell apoptosis in the peri-infarct cerebral cortex. The numbers of cleaved caspase-3-positive cells (34 ± 2.29 vs. 41.36 ± 3.38, *p* = 0.0814, Figure 6A,B) and TUNEL-positive cells (117.50 ± 9.20 vs. 110.40 ± 5.86, *p* = 0.5056, Figure 6C,D) were not significantly different between the *Celsr1*-shRNA lentivirus group and the control group. Similarly, we found that the neurological deficit score of the *Celsr1*-shRNA lentivirus group was not significantly different from that of the control group (1.67 ± 0.33 vs. 1.33 ± 0.33, *p* = 0.5185, Figure 6E). These results indicate that CELSR1 in the DG might not be neuroprotective against cell apoptosis in the peri-infarct cerebral cortex.

### 2.8. Celsr1 Knockdown in the DG Inhibited Neurogenesis but did not Affect Angiogenesis after Cerebral Ischemia

**Figure 7:**
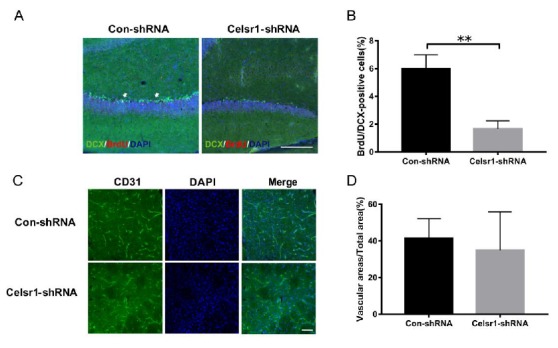
*Celsr1* knockdown in the DG promoted neurogenesis but not angiogenesis. (**A**) The number of BrdU (red)/DCX (green)-positive cells was significantly reduced in the *Celsr1*-shRNA group. Scale bar = 50 μm. (**B**) Quantitative analysis of BrdU/DCX-positive cells in the DG (*n* = 3 per group). ** *p* < 0.01. (**C**) CD31 staining in the ischemic penumbra upon Celsr1 knockdown in the DG. Scale bar = 45 μm. (**D**) Quantitative analysis of CD31-positive cells in the ischemic penumbra (*n* = 3 per group).

Next, neurogenesis and angiogenesis in the DG were explored. BrdU and DCX were used to identify neurogenesis in the DG. Compared with that in the control group, the number of BrdU/DCX-positive cells in the *Celsr1*-shRNA group was significantly reduced (6.00% ± 0.58% vs. 1.60% ± 0.33%, *p* = 0.0029, Figure 7A,B). In the peri-infarct cerebral cortex, the CD31-positive vascular area ratio in the *Celsr1*-shRNA lentivirus group was similar to that in the control group (34.91% ± 7.42% vs. 41.54% ± 3.75%, *p* = 0.4391 Figure 7C,D). These results suggest that CELSR1 in the DG promotes neurogenesis but did not affect angiogenesis in the peri-infarct cerebral cortex.

## 3. Discussion

**Figure 8:**
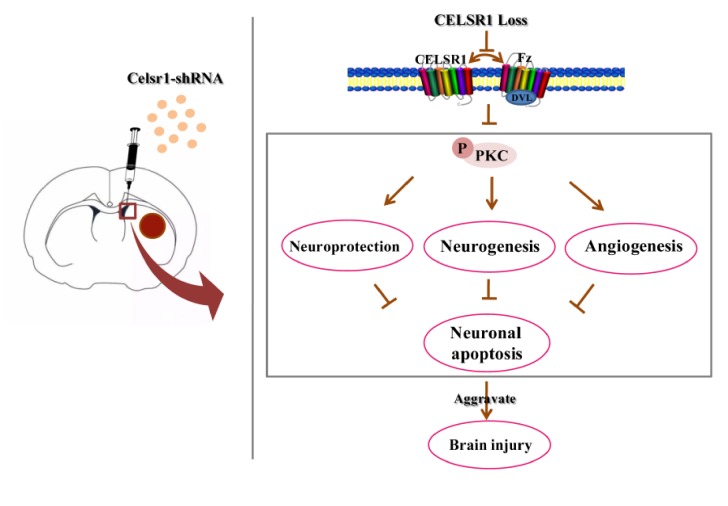
Schematic presentation of our findings showing the role of CELSR1 in the ischemic brain.

In the present study, we evaluated the neuroprotective effects of CELSR1 on cerebral ischemia in MCAO rats. The present data provide the first evidence that knocking down the expression of *Celsr1* in the SVZ increases cell apoptosis and the infarct volume and reduces neurogenesis and angiogenesis and the motor function and survival rate of MCAO rats. Moreover, the expression of p-PKC was downregulated in the SVZ and peri-infarct tissue. Knocking down *Celsr1* expression in the DG only reduced neurogenesis. Therefore, CELSR1 has a neuroprotective effect on cerebral ischemia injury by reducing cell apoptosis in the peri-infarct cerebral cortex and promoting neurogenesis and angiogenesis, mainly through the Wnt/PKC pathway (Figure 8).

In this study, we observed for the first time the neuroprotective effect of CELSR1 on cerebral ischemia injury by knocking down the expression of *Celsr1* with a lentivirus in MCAO rats. Although *Celsr1* has been identified as a susceptibility gene for ischemic stroke [15,16], the role of CELSR1 in cerebral ischemia injury is unknown. The rats were treated with a *Celsr1*-shRNA lentivirus twelve days before MCAO was performed. Our data showed that knocking down the expression of *Celsr1* in the SVZ increased the infarct volume, the percentage of apoptotic cells, motor function and the mortality rate. However, knocking down the expression of *Celsr1* in the DG did not affect the above indexes. The neuroprotective effect of CELSR1 on cerebral ischemia injury in MCAO rats was found for the first time. 

A major observation of this study was that CELSR1 promoted neurogenesis and angiogenesis in MCAO rats. The mRNA expression of *Celsr1* has been reported to be primarily confined to areas of NSCs proliferation, including the ventricular zones during brain development, the telencephalic ependymal zone, and the subgranular layer of dentate gyrus in the adult brain [11]. CELSR1 can regulate the direction of dendrite initiation sites of newborn granule cells during adult hippocampal neurogenesis [17]. Loss of function of *Celsr1* in mice results in neural progenitor fate decision defects, cortical hypoplasia and behavioral impairment [18]. CELSR1 plays an important regulatory role in the nervous system. The SVZ of the lateral ventricle and the SGZ of the hippocampal DG are two areas in which neurogenesis occurs in the mammalian adult [25,26]. In an MCAO model, ischemic injury can promote neurogenesis in the SVZ and SGZ [27,28,29]. NSCs in the SVZ can migrate to infarct-damaged areas and become newborn neurons to repair the neural circuit [30,31,32,33]. NSCs in the SGZ can migrate to the granular cell layer and become new neurons to reverse the learning and memory dysfunction induced by ischemia [34]. *Celsr1* knockdown in the SVZ or DG by shRNA lentivirus inhibited neurogenesis in MCAO rats. In human aortic endothelial cells, CELSR1 promotes cell proliferation and migration, and the formation of capillary-like structures [19]. Angiogenesis in the peri-infarct area is very important for the recovery of cerebral ischemia. *Celsr1* knockdown in the SVZ but not the DG inhibited angiogenesis. *Celsr1* knockdown in the DG did not affect angiogenesis and had no significant neuroprotective effect in MCAO rats. The reason may be that NSCs in the DG are unable to migrate to the cerebral ischemic damage areas near the cortex and striatum.

In addition to canonical Wnt signaling, noncanonical Wnt/ PCP signaling has an important regulatory role in neurogenesis, angiogenesis and cell apoptosis [17,24]. The Wnt/PKC, Wnt/JNK and Wnt/ROCK pathways are downstream pathways that are noncanonical [35,36,37]. CELSR1 is a core protein of the Wnt/PCP pathway. Thus far, the role of CELSR1 in ischemia/reperfusion-induced brain injury has not been reported. To explore the mechanism by which CELSR1 regulates neurogenesis and angiogenesis in MCAO rats, the phosphorylation levels of JNK, PKC and β-catenin were measured in SVZ and ischemic penumbra. Only the expression level of phosphorylated PKC was significantly reduced when *Celsr1* was knocked down. Based on our present study, it seems that CELSR1 regulates neurogenesis and angiogenesis in ischemia/reperfusion injury through the Wnt/PKC pathway, but not the Wnt/JNK pathway or canonical Wnt signaling.

In conclusion, this is the first report to explore the neuroprotective effect of CELSR1 on cerebral ischemia by lentiviral knockdown in MCAO rats. First, CELSR1 in the SVZ was shown to reduce neuronal apoptosis, the infarct volume and the mortality rate and restore motor function. Furthermore, neurogenesis in the SVZ and DG and angiogenesis in the ischemic penumbra were promoted by CELSR1, mainly through the Wnt/PKC pathway. These data demonstrate that CELSR1 plays a critical role in cerebral ischemia/reperfusion injury, which provides a potential target for the clinical treatment of cerebral ischemia. In this study, we verified that loss of CELSR1 protein can aggravate brain injury in rat MCAO models, but due to the limitations of lentivirus, we did not positively verify the role of CELSR1 protein (overexpression of *Celsr1*) in cerebral ischemic injury. Moreover, the study of the downstream pathways affected by CELSR1 protein is too simple, and there is no detailed study of a series of protein changes downstream of PKC. Our next work will focus on these two aspects.

## 4. Materials and Methods

### 4.1. Experimental Model

Healthy 2-month-old adult male Sprague-Dawley rats weighing 270–300 g were obtained from Vital River Laboratories (Beijing, China) and used for this study. The temperature of the feeding environment was controlled at 22 °C ± 2 °C, and the rats were kept on a 12-h light/dark cycle. All procedures were approved by the Ethics Committee on Animal Experiments of Medical School of Shandong University (No. LL-201702002, 20 May 2017).

### 4.2. Intracerebral Microinjection

After anesthesia with 10% chloral hydrate, each rat was placed into a stereotaxic frame. The head was further stabilized in a customized head mold. The right lateral ventricle was targeted at the following coordinates from bregma: −0.9 mm anterior, ±1.5 mm lateral and −3.6 mm deep. The DG of the hippocampus was targeted at the following coordinates from bregma: −3.72 mm anterior, ±2.2 mm lateral and −3.4 mm deep. The *Celsr1*-shRNA lentivirus and control lentivirus used in this study, with titers ranging from 2 × 10^8^ to 8 × 10^8^ CFU/mL, were purchased from Shanghai GeneChem Company (Shanghai, China). Two microliters of lentivirus was injected at a rate of 1 μL/min, and the needle was retained in place for 5 min following the injection. Twelve days after injection, the animals underwent MCAO for 2 h followed by reperfusion for 22 h.

### 4.3. Middle Cerebral Artery (MCA) Occlusion Model

Rats were subjected to MCAO based on a published protocol [38]. Briefly, rats were anesthetized with 10% chloral hydrate. The right common carotid artery (CCA), external carotid artery (ECA), and internal carotid artery (ICA) were isolated. A nylon filament (0.28 mm in diameter) with an expanded tip was gently advanced from the CCA into the lumen of the ICA. The tip of the filament was positioned at the origin of the middle cerebral artery (MCA). The right MCA was occluded with the filament for 2 h, and then the filament was withdrawn to allow 22 h of reperfusion. During recovery from the anesthesia, the animals were returned to their home cages.

### 4.4. Examination of Neurological Deficits

Behavioral assays were performed as previously described [1,38]. Scales from zero to four were used to assess the effects of MCAO on neurological behavior. After 22-h, 46-h or 70-h reperfusion, the rats were scored according to the guidelines.

### 4.5. Evaluation of Infarct Volume

Three days after MCAO, the infarct area was measured by 2% TTC (Sigma Chemical Co., St. Louis, MO, USA) staining as previously described [1]. The infarct area of each slice was measured with NIH’s ImageJ software, version 1.46 (Bethesda, MA, USA), as previously described. 

### 4.6. BrdU Labeling

After MCAO 72 h, the rats were injected intraperitoneally with 50 mg/kg BrdU (Sigma). The rats were perfused 2 h after the injection (SVZ: Con-shRNA group, n = 5; *Celsr1*-shRNA group, n = 4; DG: n = 3 in each group). Rats were transcardially perfused with normal saline followed by 4% paraformaldehyde. The brains were removed and fixed in 4% paraformaldehyde.

### 4.7. Immunohistochemistry

Serial sagittal or coronal (40 µm) sections were cut with a cryostat and stored at −80 °C. For immunofluorescence staining, the sections were incubated overnight with primary antibody at 4 °C. The primary antibodies used were mouse monoclonal anti-CD31 (1:50; Proteintech Group, Chicago, IL, USA), sheep monoclonal anti-BrdU (1:500; Cell Signaling Technology, Danvers, MA, USA) and rabbit monoclonal anti-cleaved caspase-3 (1:500; Cell Signaling Technology, Danvers, MA, USA). The secondary antibodies used were Alexa Fluor 488-conjugated IgG (1:1000; Invitrogen, Carlsbad, CA, USA) and Alexa Fluor 594-conjugated IgG (1:1000; Invitrogen, Carlsbad, CA, USA). The nuclei of the cells were counterstained with 4,6-diamidino-2-phenylindole (DAPI).

### 4.8. Western Blot Analysis

Three days after MCAO in rats, tissues from the SVZ and ischemic penumbra were harvested. The tissues were lysed in lysis buffer containing 1% protease inhibitor and 1% phosphatase inhibitor followed by centrifugation for 15 min at 14,000 rpm. The total protein was separated by SDS-PAGE and transferred to a nitrocellulose membrane. The membrane was blocked and incubated with an appropriate primary antibody and secondary antibody. The following antibodies were used: mouse anti-GAPDH (1:5,000, CST, Danvers, MA, USA), rabbit anti-PKC (1:500, Wanleibio, Shen Yang, China), rabbit anti-JNK (1:500, Wanleibio, Shen Yang, China), rabbit anti-β-catenin (1:10,000, Proteintech Group, Chicago, IL, USA), rabbit anti-p-PKC (1:10,00, CST, Danvers, MA, USA), rabbit anti-p-JNK (1:500, Wanleibio, Shen Yang, China), and horseradish peroxidase (HRP)-conjugated secondary antibodies (1:10,000; Millipore, Billerica, MA, USA).

### 4.9. Quantitative RT-PCR

Tissues from different areas of the brain were harvested from the MCAO group and the sham operation group. TRIzol was used to extract total RNA, and the RNA was reverse transcribed into cDNA. The resulting cDNA was amplified by PCR in the presence of oligonucleotide primer pairs designed to target *Celsr1* cDNA and β-actin. The primers used for PCR were as follows: β-actin fo: gag agg gaa atc gtg cgt gac, re: cat acc cag gaa gga agg ct; *Celsr1* fo: gcc agt ttg ctg ttg ctc, re: gac agg ctt gct tcg ttc. The PCR conditions were 10 min at 95 °C, 39 cycles at 95 °C for 15 s, 60 °C for 30 s and 72 °C for 30 s, followed by incubation at 72 °C for 10 min and maintenance at 4 °C.

### 4.10. TUNEL Staining

Three days after MCAO, brains were serially sliced in coronal sections at a thickness of 40 μm with a cryostat. The sections were assessed by TUNEL assay. The kit was purchased from Promega (Madison, WI, USA), and the experiment was conducted according to the manufacturer’s protocol.

### 4.11. Cell Transfection

HEK293T cells were seeded into 6-well plates and cultured at 37 °C and 5% CO_2_ for 24 h. Then, the old culture medium was replaced with fresh medium containing 4 μL of *Celsr1*-shRNA lentivirus with GFP. After 48 h of viral infection, the cells were harvested, and RNA was extracted.

### 4.12. Statistical Analysis

The results are presented as the mean ± SEM. Statistical analysis of differences was conducted using Student’s *t*-tests. A statistically significant difference was set at *p* < 0.05.

## 5. Conclusions

CELSR1 has neuroprotective effects in cerebral ischemia/reperfusion injury, and the protective mechanism of CELSR1 seems mainly through the Wnt/PKC pathway. These findings suggest that CELSR1 may be a potential target for the clinical treatment of cerebral ischemia. 

## Abbreviations

CELSR1	Cadherin epidermal growth factor (EGF) laminin G (LAG) seven-pass G-type receptor 1
MCAO	middle cerebral artery occlusion
SVZ	subventricular zone
tPA	tissue plasminogen activator
SGZ	subgranular zone
NSCs	neural stem cells
PCP	planar cell polarity
GFP	green fluorescent protein
DG	dentate gyrus
CCA	common carotid artery
ECA	external carotid artery
ICA	internal carotid artery
MCA	middle cerebral artery
TTC	triphenyltetrazolium chloride
TUNEL	TdT-mediated dUTP-biotin nick end labeling
